# Supplementary material for: Cross-institutional dental electronic health record entity extraction via generative artificial intelligence and synthetic notes
Source: JAMIA Open. 2025 Jun 28;8(3):ooaf061. doi: 10.1093/jamiaopen/ooaf061 (PMC12205731; doi:10.1093/jamiaopen/ooaf061)
Supplement: ooaf061_Supplementary_Data [file ooaf061_supplementary_data.docx]

**Cross-Institutional Dental EHR Entity Extraction via Generative AI and Synthetic Notes**

Yao-Shun Chuang^1^, Chun-Teh Lee^2^, Guo-Hao Lin^3^, Ryan Brandon^4^, Xiaoqian Jiang^1^, Muhammad F. Walji^1,2^, Bunmi Tokede^2^

^1^ McWilliams School of Biomedical Informatics, The University of Texas Health Science Center at Houston, 7000 Fannin Street, Houston, TX 77030

^2^ The University of Texas Health Science Center at Houston School of Dentistry, 7500 Cambridge St, Houston, TX 77054

^3^ Postgraduate Periodontics Program, School of Dentistry, University of California San Francisco, 707 Parnassus Avenue, D-3015, San Francisco, CA 94143, USA

^4^ Willamette Dental Group and Skourtes Institute, 5935 SE Alexander St, Hillsboro, OR 97123

**Supplementary Appendix A**

| {  "role": "user",  "content": "As a dentist, your task is to perform an oral examination on a patient and document any instances of Periodontitis (including its Disease (Periodontitis), Stage (I, II, III, IV), Grade (A, B, C), and Extent (Generalized, Localized). "  }, {  "role": "user",  "content": "Generate one row of values in a table with 4 columns, including Disease (Periodontitis), Stage (I, II, III, IV), Grade (A, B, C), and Extent (Generalized, Localized). Then create a clinical note that explicitly contains these values (Disease, Stage, Grade, and Extent) and is similar in style to the example provided below. "  }, {  "role": "user",  "content": "The example note is: … "  } |
| --- |
| {  "role": "user",  "content": "As a dentist, your task is to perform an oral examination on a patient and document instances including its Disease (Gingivitis), Extent (Generalized, Localized), and Subtype (intact periodontium, reduced periodontium, past or stable periodontitis, past periodontitis, stable periodontitis, non-periodontitis)."  }, {  "role": "user",  "content": "Generate one row of values in a table with 3 columns, including Disease (Gingivitis), Extent (Generalized, Localized), and Subtype (intact periodontium, reduced periodontium, past or stable periodontitis, past periodontitis, stable periodontitis, non-periodontitis). Then create a clinical note that explicitly contains these values (Disease, Extent, and Subtype) and is similar in style to the example provided below."  }, {  "role": "user",  "content": "The example note is: … "  } |
| {  "role": "user",  "content": " As a dentist, your task is to perform an oral examination on a patient and document any instances of normal periodontal tissue (including its Disease (periodontal health, clinical gingival health, gingival health, clinical gingival healthy, gingival healthy, normal periodontal tissue), and Subtype (intact periodontium, reduced periodontium, past or stable periodontitis, past periodontitis, stable periodontitis, non-periodontitis) )."  }, {  "role": "user",  "content": "Generate one row of values in a table with 2 columns, including Disease (periodontal health, clinical gingival health, gingival health, clinical gingival healthy, gingival healthy, normal periodontal tissue), and Subtype (intact periodontium, reduced periodontium, past or stable periodontitis, past periodontitis, stable periodontitis, non-periodontitis). Then create a clinical note that explicitly contains these values (Disease, and Subtype) and is similar in style to the example provided below."  }, {  "role": "user",  "content": "The example note is: … "  } |

**Fig. 1.** The example prompts for generating synthetic notes for each periodontal status using ChatGPT, ordered from top to bottom: periodontitis, gingivitis, and health.
